# Supplementary material for: Accurate imputation of inversions in human genomes using different algorithms and data sources
Source: NAR Genom Bioinform. 2026 Aug 25;8(3):lqag094. doi: 10.1093/nargab/lqag094 (PMC13504282; doi:10.1093/nargab/lqag094)
Supplement: Supplementary file 1 [file lqag094_supplemental_files.zip › SupplementaryFigures_v4_Submitted.pdf]

Supplementary Figures

Accurate imputation of inversions in human genomes using different algorithms and data sources

Illya Yakymenko[1,2 †], Adrià Mompart[1 †], Mario Cáceres[1,2,3 *]

1. Research Program on Biomedical Informatics (GRIB), Hospital del Mar Research Institute, Barcelona 08003, Spain.

2. Institut de Biotecnologia i de Biomedicina, Universitat Autònoma de Barcelona, Bellaterra 08193, Barcelona, Spain.

3. ICREA, Barcelona 08010, Spain.

[†] These authors contributed equally to this work.

* Corresponding author. mcaceres@icrea.cat

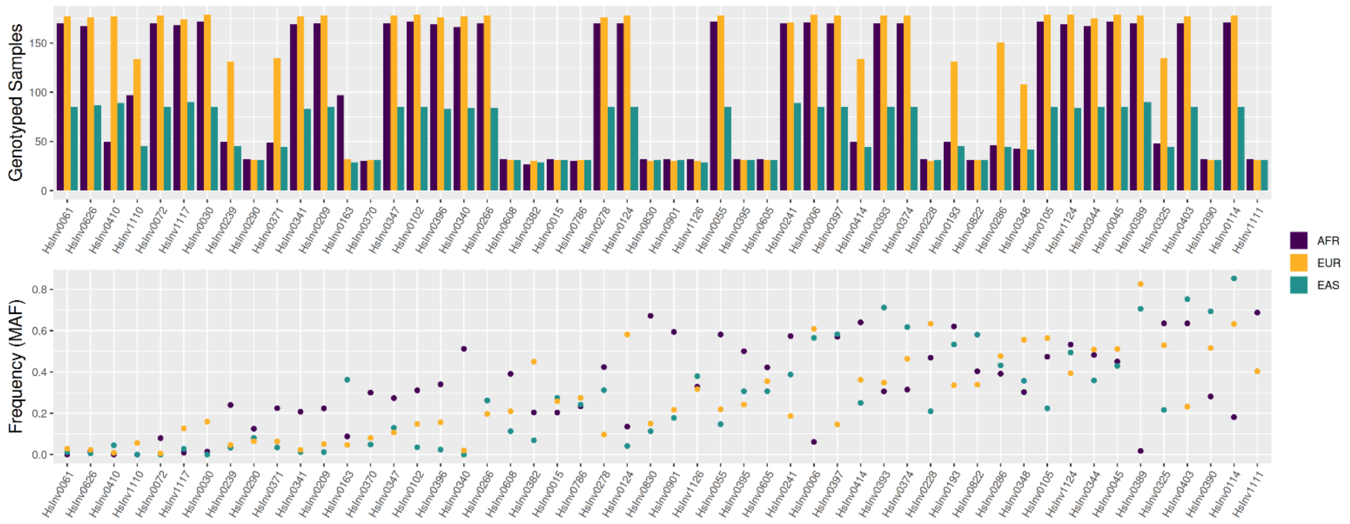

Supplementary Figure S1. Summary of available information for the 52 inversions analyzed, including the number of experimentally genotyped samples (top) and the minor allele frequency (MAF, bottom) in the African (AFR), European (EUR) and East-Asian super-populations.

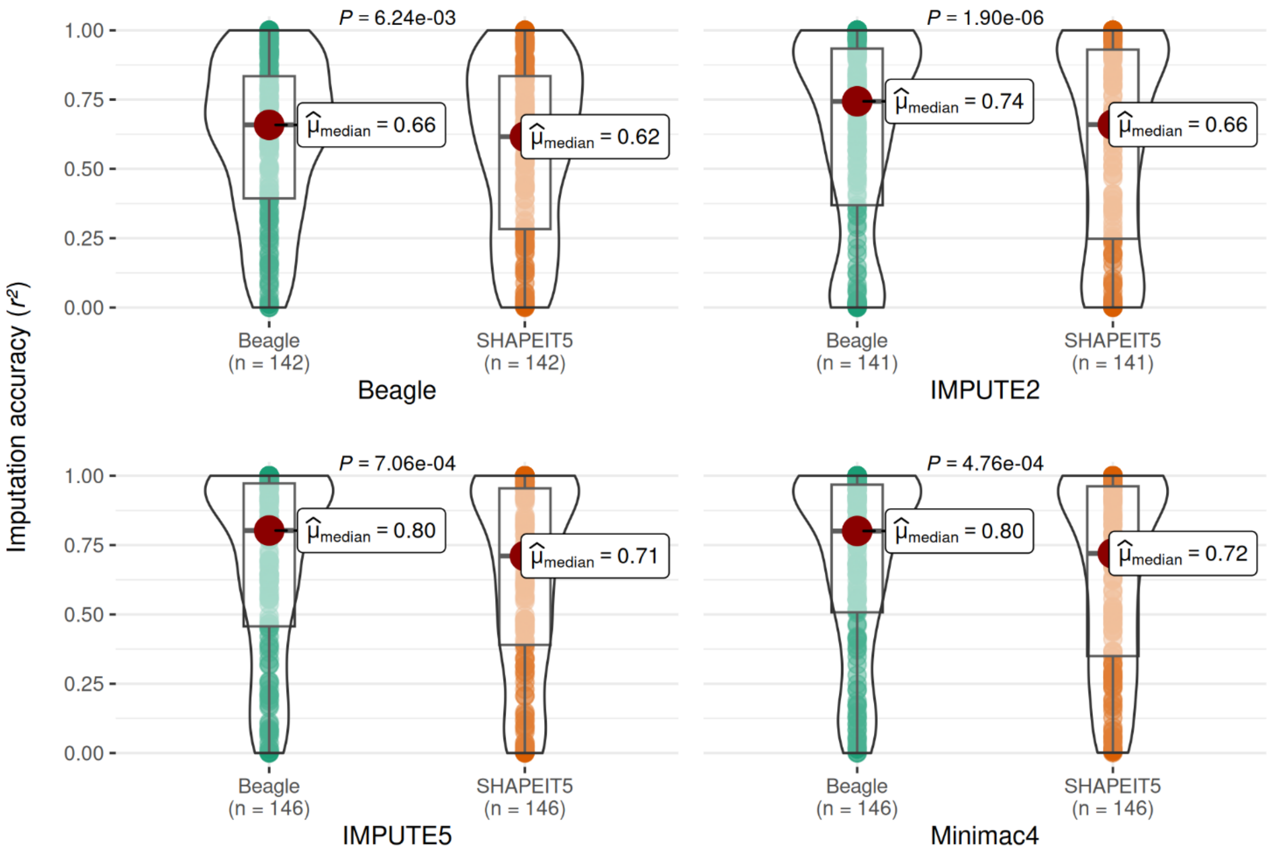

Supplementary Figure S2. Comparison of inversion imputation results with different phasing and imputation programs. Violin plots represent the imputation accuracy (r^2) of the 52 analyzed inversions in each of the three super-populations with four imputation programs, after inversion phasing using Beagle (green, left) or SHAPEIT5 (orange, right). Inversions included in the analysis are those including the two orientations in a population in both phasing programs, in which the r^2 can be calculated (indicated by n), and the median r^2 value across inversions and populations is shown inside the rectangle (μ). The *P* value of the difference in imputation accuracy between Beagle and SHAPEIT5 is shown on top of each comparison and was calculated by a paired Wilcoxon signed-rank test.

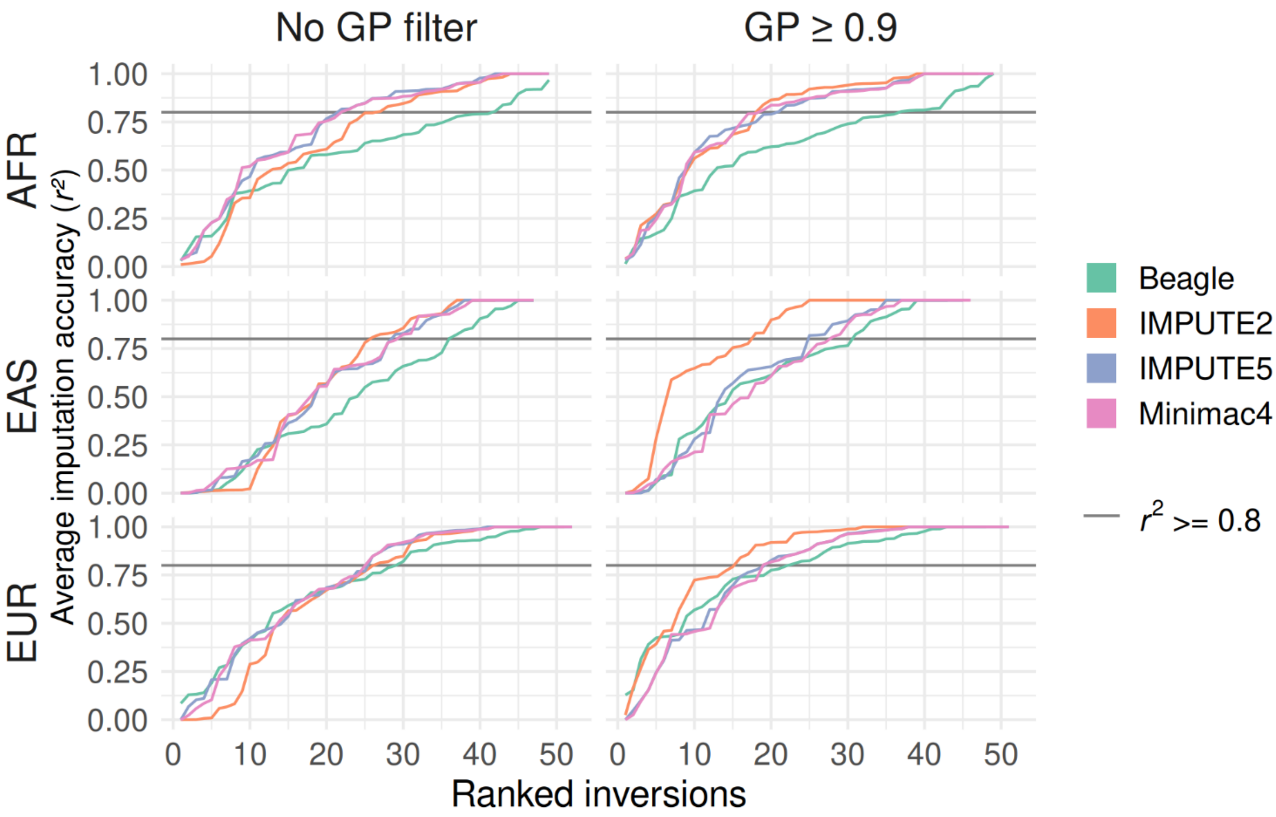

Supplementary Figure S3. Distribution of inversion imputation accuracy (r^2) for four of the programs tested. Inversions are ranked according to the r^2 across all samples of each population (Y axis), from the lowest to the highest value per method (including inversions in which r^2 cannot be calculated due to lack of imputed minor alleles as 0). Both the original r^2 values (left) and those after filtering out imputed genotypes with probability (GP) lower than 0.9 (right) are represented. The faster the r^2 = 0.8 imputability threshold is reached (represented by a solid line), indicates that there is a higher number of imputable inversions. Conversely, with Beagle a lower proportion of imputable inversions is obtained in all cases. IMPUTE2 shows better performance than other methods with the 0.9 GP filter, although this is based on less genotypes for many inversions and a smaller number of easier to impute inversions.

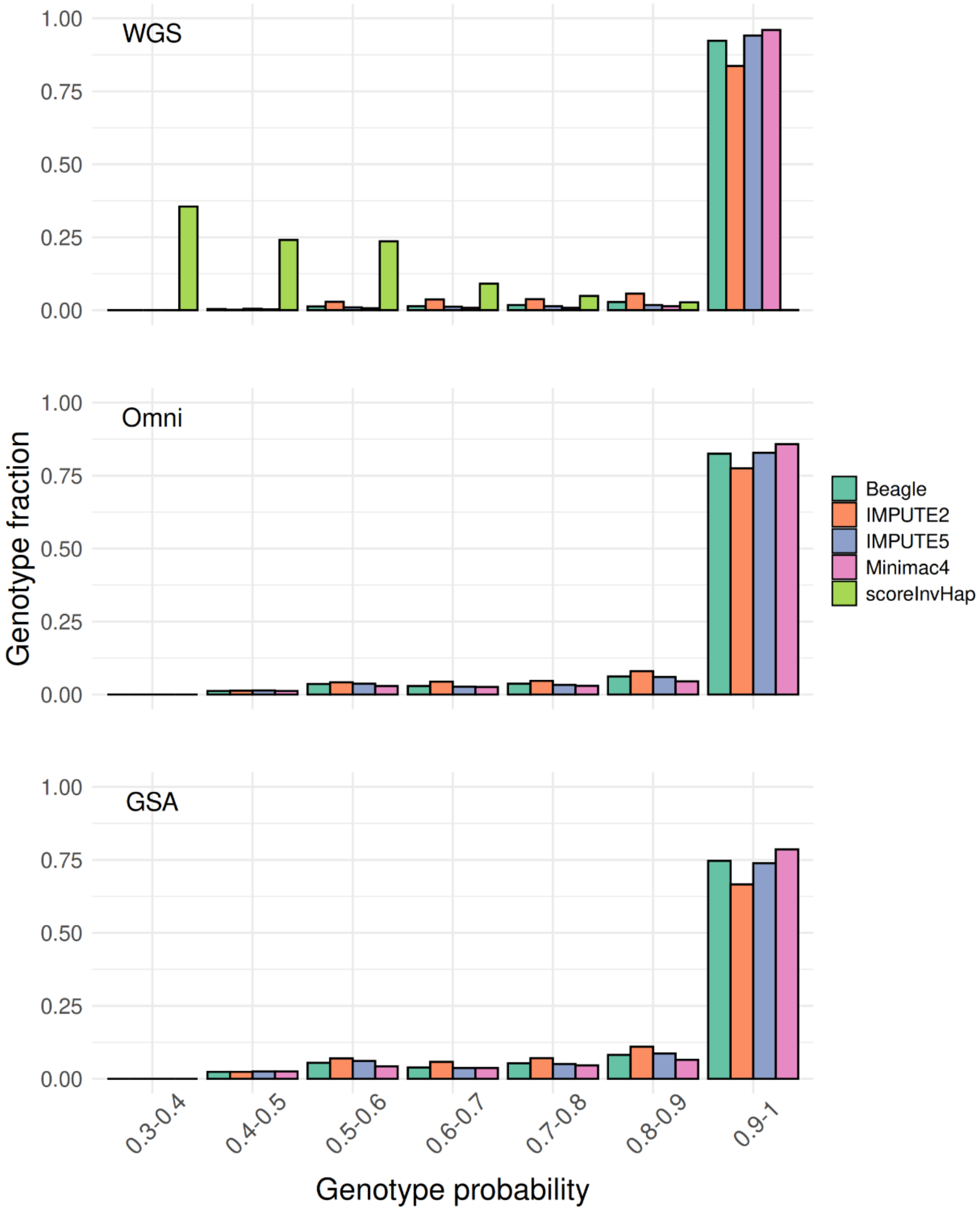

Supplementary Figure S4. Histogram of the distribution of genotype probability (GP) values of imputed genotypes obtained by each program using nucleotide variation data from whole genome sequence (WGS) or Omni and GSA SNP genotyping arrays. Fraction of genotypes is calculated in GP bins of 0.1 by combining all samples from different populations and inversions together.

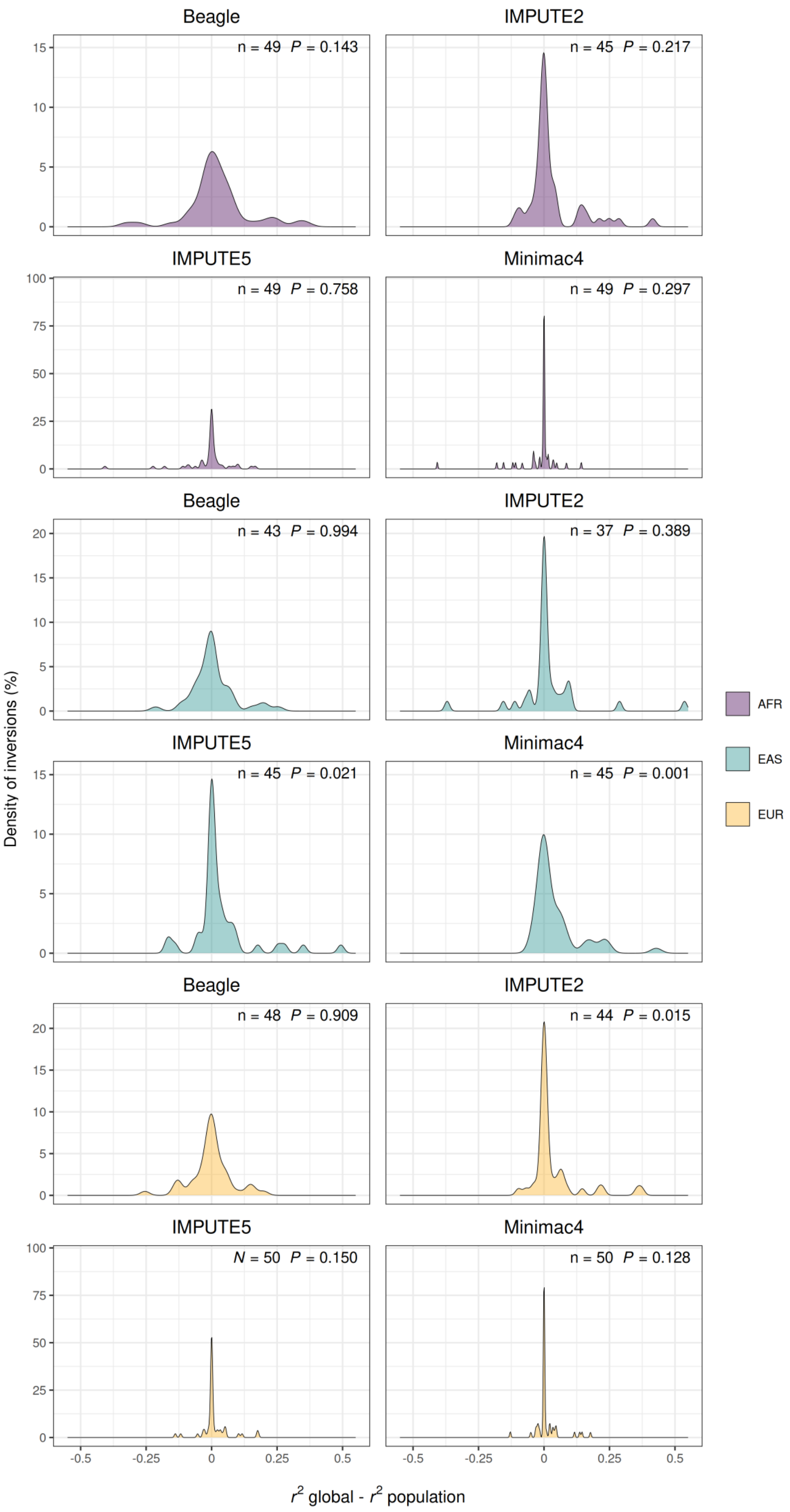

Beagle
n = 49 P = 0.143
IMPUTE2
n = 45 P = 0.217
IMPUTE5
n = 49 P = 0.758
Minimac4
n = 49 P = 0.297
Beagle
n = 43 P = 0.994
IMPUTE2
n = 37 P = 0.389
IMPUTE5
n = 45 P = 0.021
Minimac4
n = 45 P = 0.001
Beagle
n = 48 P = 0.909
IMPUTE2
n = 44 P = 0.015
IMPUTE5
N = 50 P = 0.150
Minimac4
n = 50 P = 0.128
Density of inversions (%)
r2 global - r2 population
AFR
EAS
EUR

Supplementary Figure S5. Detailed comparison of imputation accuracy (r^2) obtained by using different reference panels with four imputation programs. Each graph shows the density distribution across all analyzed inversions (Y axis) of the difference in r^2 of the global minus the population reference panel (X axis) separately for each population and imputation program, with the three populations represented in different colors. The number of inversions analyzed in each case (n) and the *P* value of a Wilcoxon paired signed-rank test to compare the r^2 of the two reference panels are shown inside the graphs.

A

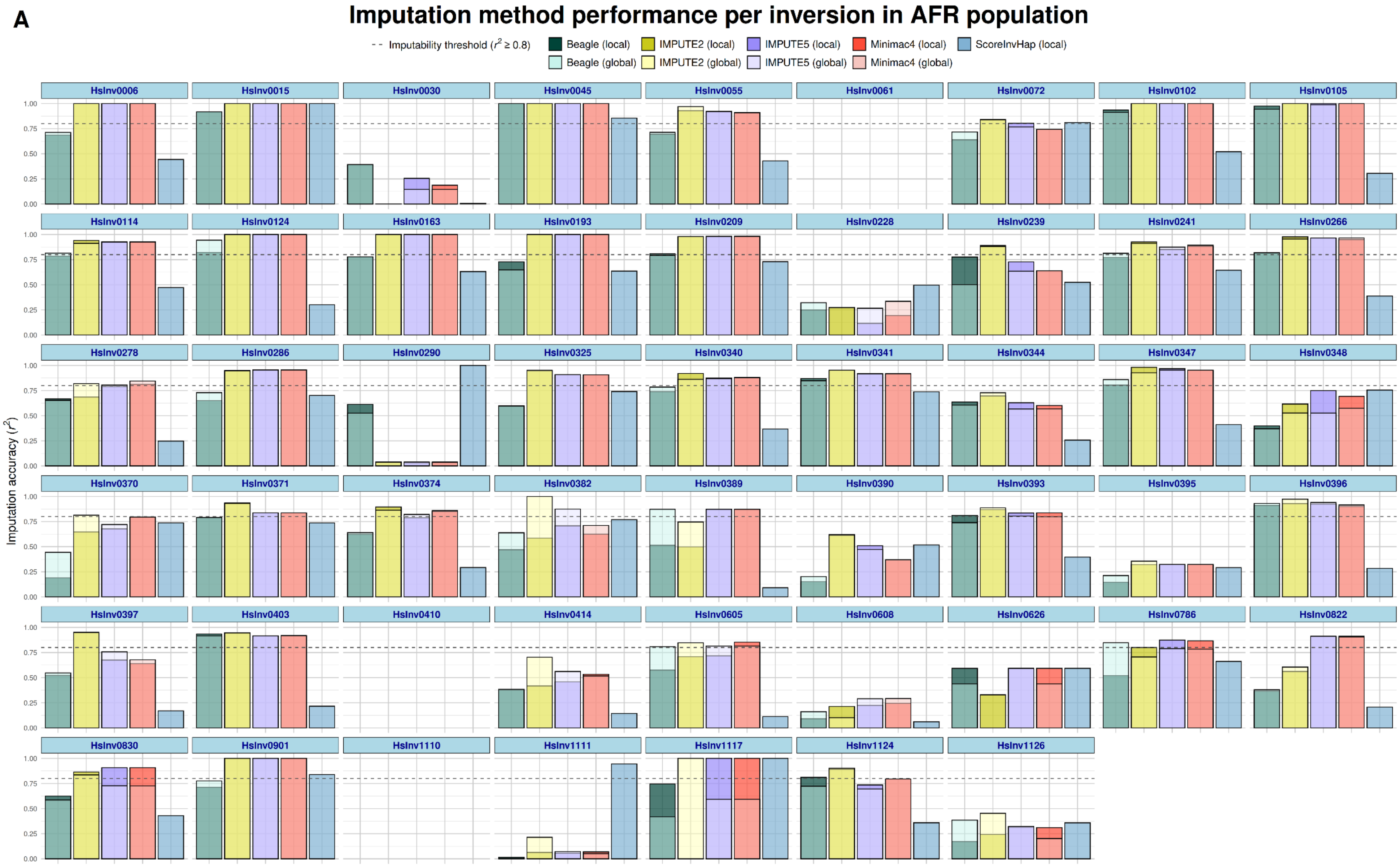

Imputation method performance per inversion in AFR population
Imputability threshold ($r^2 \geq 0.8$)
Beagle (local)
IMPUTE2 (local)
IMPUTE5 (local)
Minimac4 (local)
ScoreInvHap (local)
Beagle (global)
IMPUTE2 (global)
IMPUTE5 (global)
Minimac4 (global)
Imputation accuracy (r^2)
HsInv0006
HsInv0015
HsInv0030
HsInv0045
HsInv0055
HsInv0061
HsInv0072
HsInv0102
HsInv0105
HsInv0114
HsInv0124
HsInv0163
HsInv0193
HsInv0209
HsInv0228
HsInv0239
HsInv0241
HsInv0266
HsInv0278
HsInv0286
HsInv0290
HsInv0325
HsInv0340
HsInv0341
HsInv0344
HsInv0347
HsInv0348
HsInv0370
HsInv0371
HsInv0374
HsInv0382
HsInv0389
HsInv0390
HsInv0393
HsInv0395
HsInv0396
HsInv0397
HsInv0403
HsInv0410
HsInv0414
HsInv0605
HsInv0608
HsInv0626
HsInv0786
HsInv0822
HsInv0830
HsInv0901
HsInv1110
HsInv1111
HsInv1117
HsInv1124
HsInv1126
0.00
0.25
0.50
0.75
1.00

B

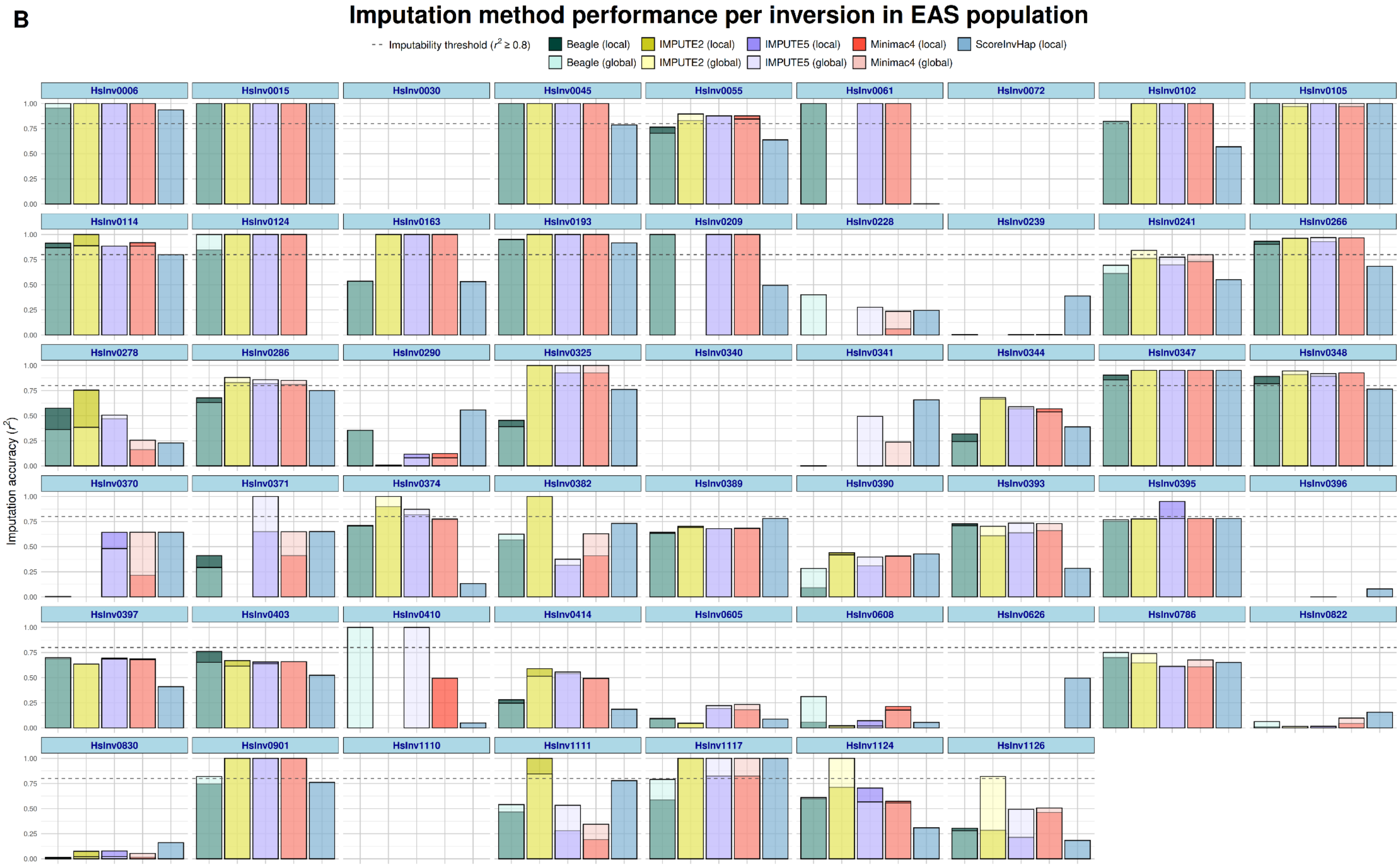
Imputation method performance per inversion in EAS population
Imputability threshold ($r^2 \geq 0.8$)
Beagle (local)
IMPUTE2 (local)
IMPUTE5 (local)
Minimac4 (local)
ScoreInvHap (local)
Beagle (global)
IMPUTE2 (global)
IMPUTE5 (global)
Minimac4 (global)
Imputation accuracy (r^2)
0.00
0.25
0.50
0.75
1.00
HsInv0006
HsInv0015
HsInv0030
HsInv0045
HsInv0055
HsInv0061
HsInv0072
HsInv0102
HsInv0105
HsInv0114
HsInv0124
HsInv0163
HsInv0193
HsInv0209
HsInv0228
HsInv0239
HsInv0241
HsInv0266
HsInv0278
HsInv0286
HsInv0290
HsInv0325
HsInv0340
HsInv0341
HsInv0344
HsInv0347
HsInv0348
HsInv0370
HsInv0371
HsInv0374
HsInv0382
HsInv0389
HsInv0390
HsInv0393
HsInv0395
HsInv0396
HsInv0397
HsInv0403
HsInv0410
HsInv0414
HsInv0605
HsInv0608
HsInv0626
HsInv0786
HsInv0822
HsInv0830
HsInv0901
HsInv1110
HsInv1111
HsInv1117
HsInv1124
HsInv1126

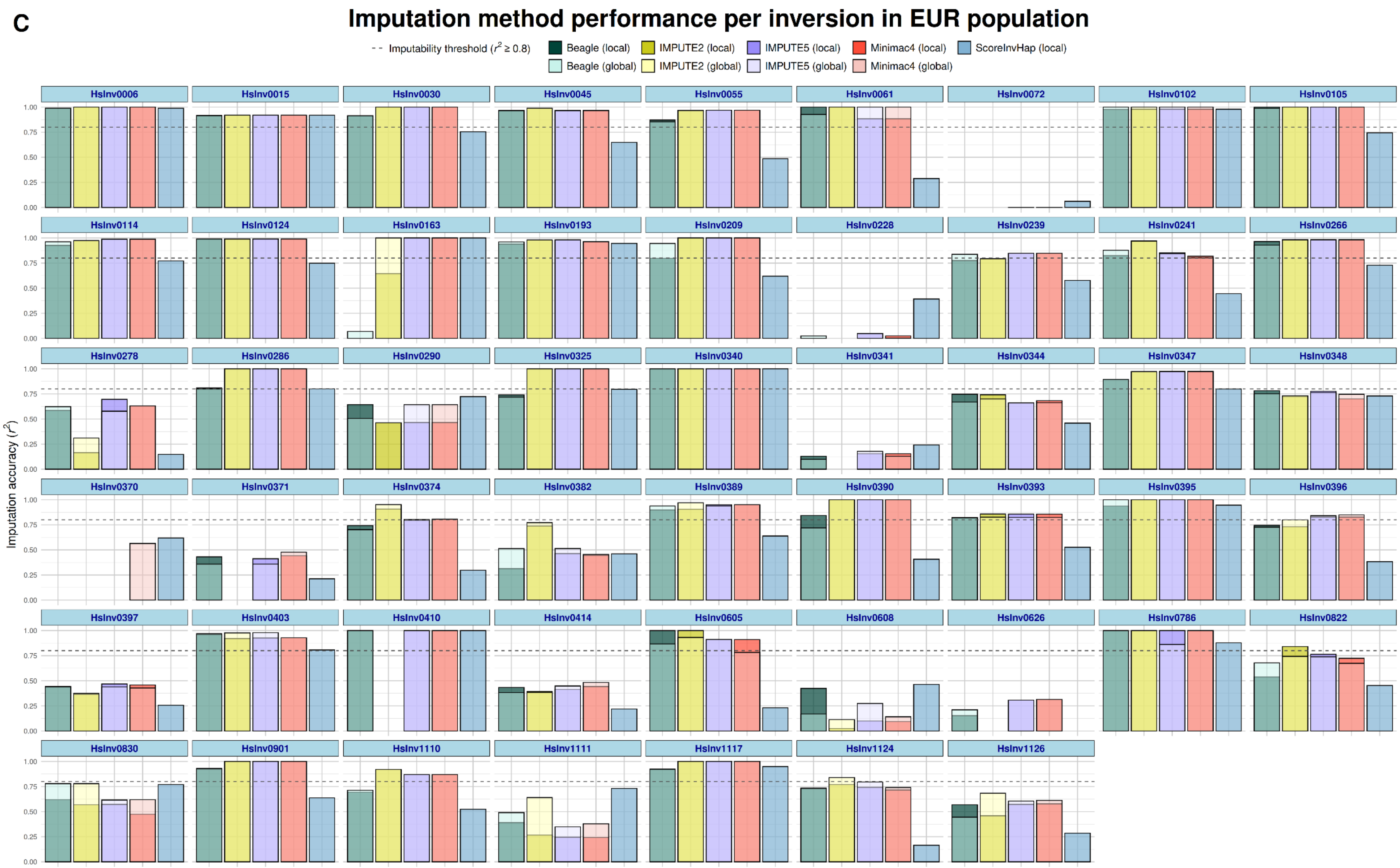
C
Imputation method performance per inversion in EUR population
Imputability threshold (r^2 ≥ 0.8)
Beagle (local)
IMPUTE2 (local)
IMPUTE5 (local)
Minimac4 (local)
ScoreInvHap (local)
Beagle (global)
IMPUTE2 (global)
IMPUTE5 (global)
Minimac4 (global)
Imputation accuracy (r^2)
0.00
0.25
0.50
0.75
1.00
HsInv0006
HsInv0015
HsInv0030
HsInv0045
HsInv0055
HsInv0061
HsInv0072
HsInv0102
HsInv0105
HsInv0114
HsInv0124
HsInv0163
HsInv0193
HsInv0209
HsInv0228
HsInv0239
HsInv0241
HsInv0266
HsInv0278
HsInv0286
HsInv0290
HsInv0325
HsInv0340
HsInv0341
HsInv0344
HsInv0347
HsInv0348
HsInv0370
HsInv0371
HsInv0374
HsInv0382
HsInv0389
HsInv0390
HsInv0393
HsInv0395
HsInv0396
HsInv0397
HsInv0403
HsInv0410
HsInv0414
HsInv0605
HsInv0608
HsInv0626
HsInv0786
HsInv0822
HsInv0830
HsInv0901
HsInv1110
HsInv1111
HsInv1117
HsInv1124
HsInv1126

Supplementary Figure S6. Individual inversion imputation results in the three studied populations. (**A–C**) Graphs show the imputation accuracy (r^2) of each inversion with the five tested programs (GP ≥ 0.9 filtering, except for scoreInvHap) using a population specific (local, dark color) or global (light color) reference panel in African (AFR, **A**), East-Asian (EAS, **B**) and European (EUR, **C**) populations. Dashed line indicates the r^2 ≥ 0.8 threshold to consider an inversion as imputable.

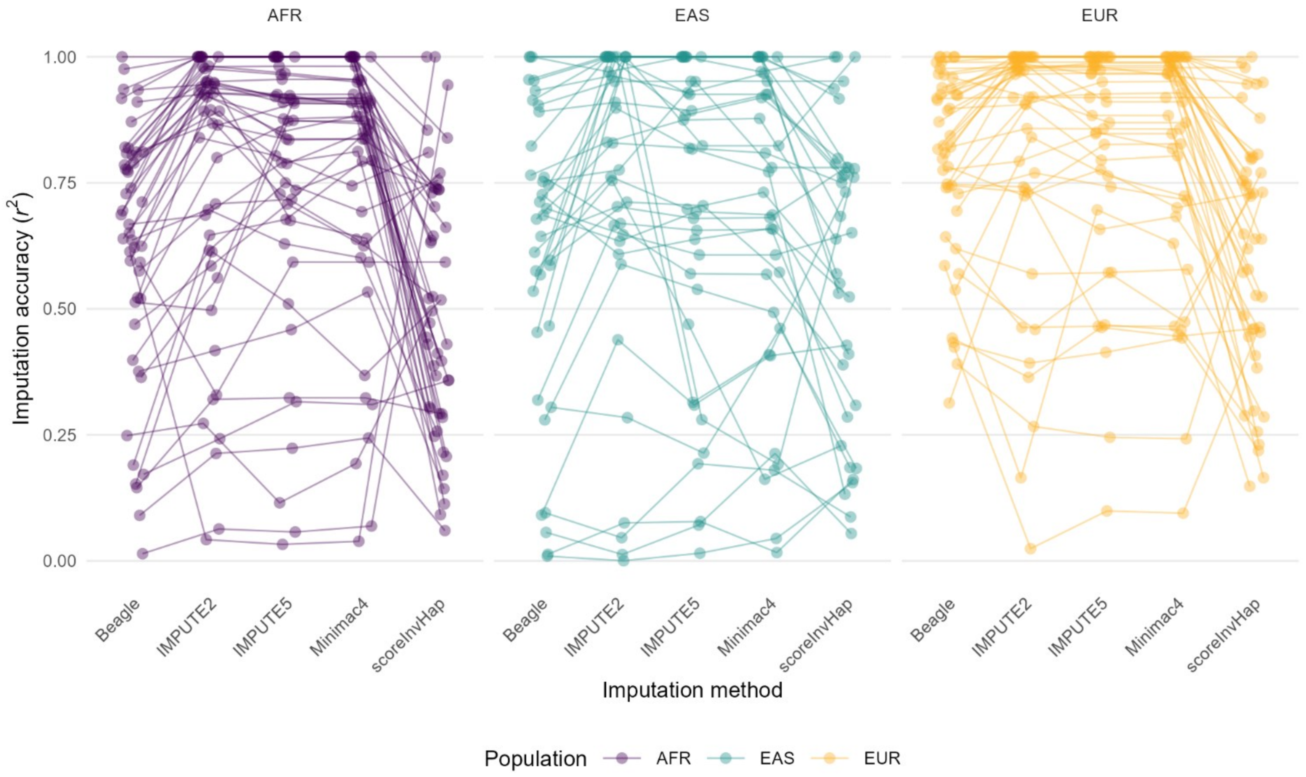

Supplementary Figure S7. Comparison of inversion imputation accuracy (r^2) of different imputation methods. Slope chart showing the variation of r^2 values of individual inversions across five distinct methods (Beagle, IMPUTE2, IMPUTE5, Minimac4, and scoreInvHap) represented as solid dots connected by lines, stratified by super-population (AFR, EAS, and EUR). A genotype probability (GP) filtering of ≥0.9 was applied to Beagle, IMPUTE2, IMPUTE5 and Minimac4. Inversions lacking a r^2 estimate for any of the five tools were excluded from that specific population comparison. Relatively consistent results were obtained for most methods in the three populations, except for a lower imputation accuracy in Beagle and especially scoreInvHap for many inversions.

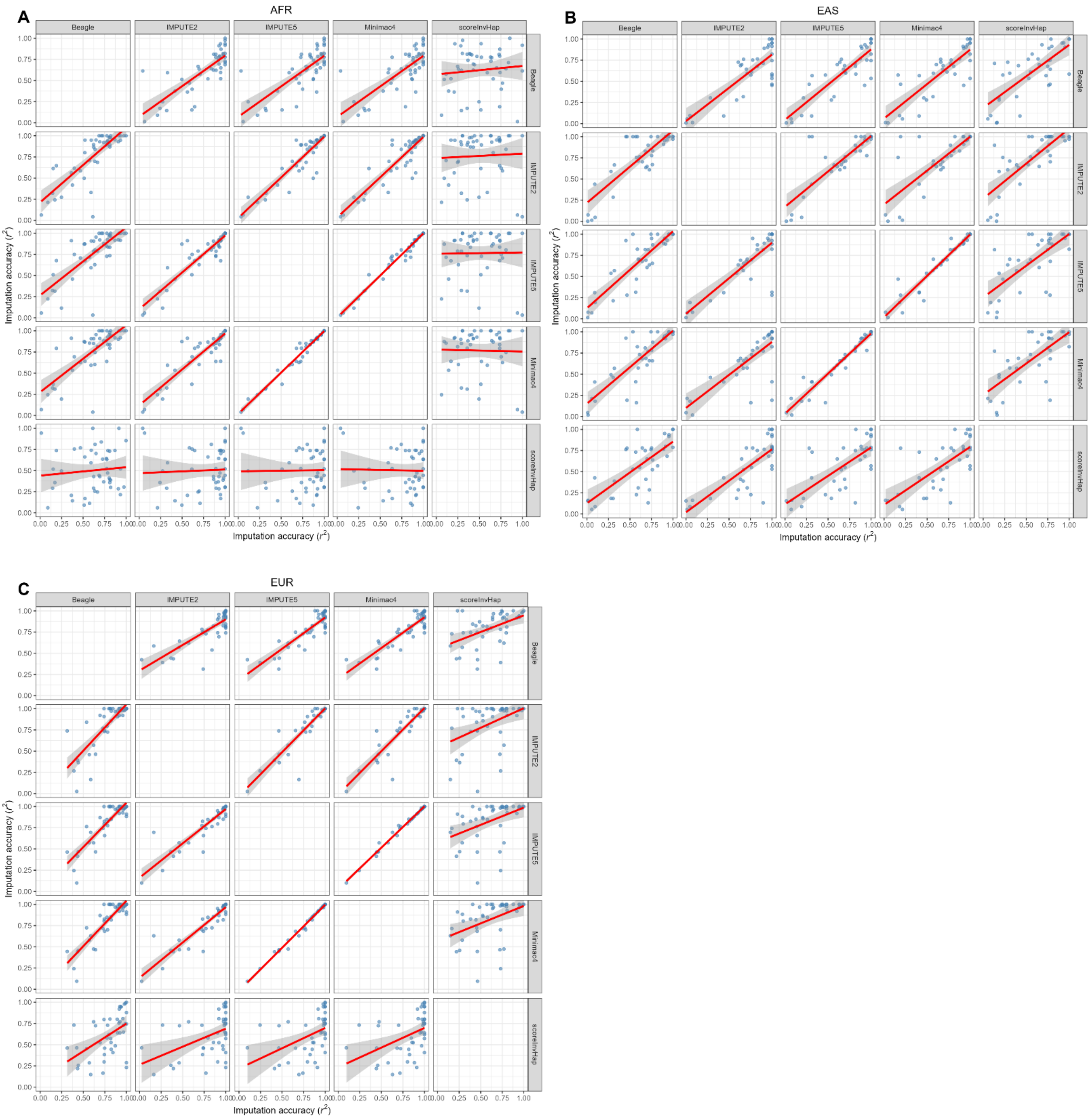

Supplementary Figure S8. Pairwise comparison of inversion imputation accuracy across methods in different populations. (**A–C**) Scatter plot matrices comparing the imputation accuracy (r^2) between each pair of methods for AFR (A), EAS (B), and EUR (C) super-populations. Each data point represents a single inversion, and X and Y axes correspond to the r^2 of the respective methods indicated on the top and the right of the graphs. An ordinary least squares linear regression is fitted on each pairwise comparison (solid red lines), with shaded regions indicating the 95% confidence

intervals. A genotype probability (GP) filtering of ≥0.9 was applied to Beagle, IMPUTE2, IMPUTE5 and Minimac4, and only inversions in which the r^2 could be calculated in the two compared methods were included in the graph.

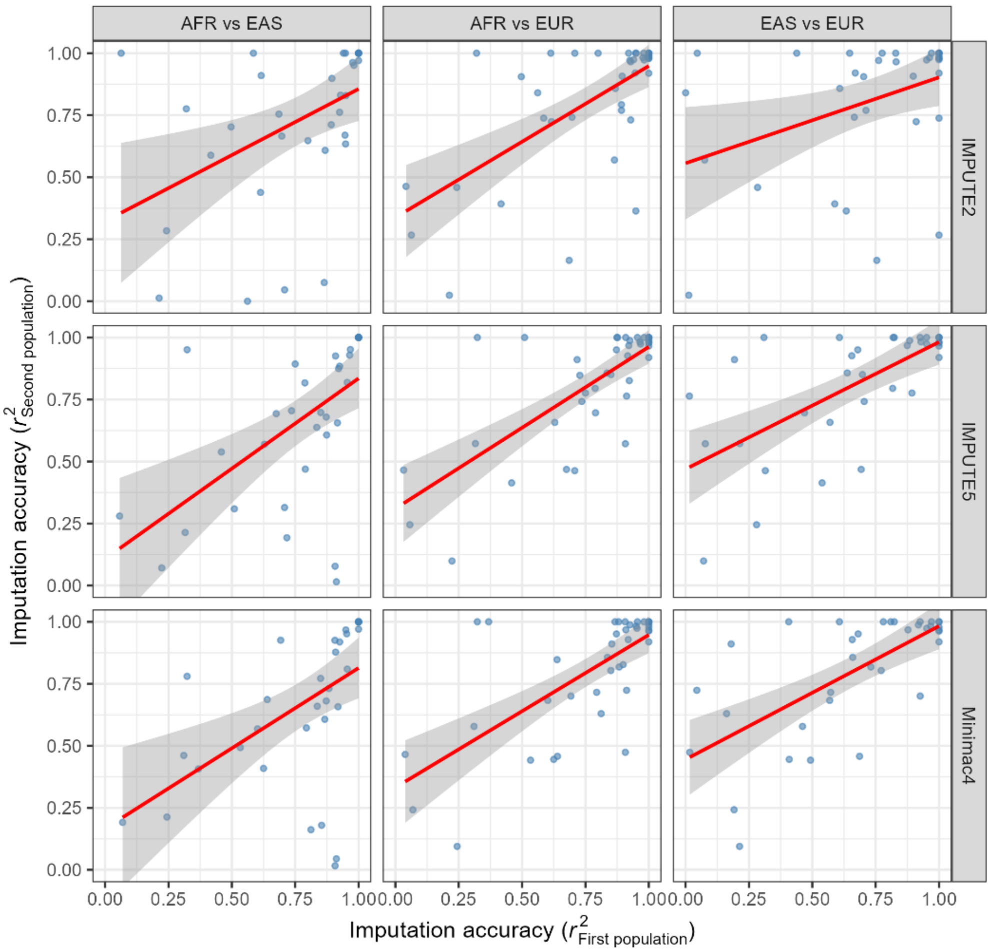

Supplementary Figure S9. Pairwise comparison of inversion imputation accuracy between populations across different methods in different populations. Scatter plots comparing the imputation accuracy (r^2) of individual inversions between pairs of super-populations (AFR, EAS, and EUR; displayed in columns) across the three best performing imputation methods: IMPUTE2, IMPUTE5, and Minimac4 (displayed in rows). Each data point represents a single inversion, and X and Y axes correspond to the r^2 of the respective populations indicated on the top of the graphs, for each of the assayed methods (indicated in the right side). An ordinary least squares linear regression is fitted on each pairwise comparison (solid red lines), with shaded regions indicating the 95% confidence intervals. A genotype probability (GP) filtering of ≥0.9 was applied to all methods, and only inversions in which the r^2 could be calculated in the two compared populations were included in the graph.
